# Evaluation of chest radiography, *lytA* real-time PCR, and other routine tests for diagnosis of community-acquired pneumonia and estimation of possible attributable fraction of pneumococcus in northern Togo

**DOI:** 10.1017/S0950268816002211

**Published:** 2016-11-17

**Authors:** A. BLAKE, B. M. NJANPOP-LAFOURCADE, J. N. TELLES, A. RAJOHARISON, M. S. MAKAWA, K. AGBENOKO, S. TAMEKLOE, J. E. MUELLER, H. TALL, B. D. GESSNER, G. PARANHOS-BACCALÀ, J. C. MOÏSI

**Affiliations:** 1 Agence de Médecine Préventive, Paris, France; 2 Fondation Mérieux – Laboratoire des Pathogènes Emergents, Lyon, France; 3 Ministère de la Santé, Lomé, Togo; 4 EHESP French School of Public Health, Sorbonne Paris Cité, Rennes France; 5 Institut Pasteur, Paris, France

**Keywords:** Aetiology, chest radiography, latent class analysis, pneumonia, *Streptococcus pneumoniae*

## Abstract

*Streptococcus pneumoniae* (Spn) is a leading cause of community-acquired pneumonia (CAP), yet existing diagnostic tools remain inadequate. We aimed to evaluate laboratory and radiological methods for detecting pneumococcal aetiology in CAP patients and to estimate Spn prevalence in this group. All-aged patients hospitalized with clinically defined CAP in northern Togo were enrolled during 2010–2013. Latent class analysis pooled results of semi-automated blood culture (SABC), whole blood *lytA* real-time polymerase chain reaction (rt-PCR), serum C-reactive protein (CRP), and chest radiography (CXR) and categorized patients as likely pneumococcal or non-pneumococcal CAP. We enrolled 1684 patients; 1501 had results for all tests. CXR, SABC, *lytA* rt-PCR and CRP >71·2 mg/l had sensitivities of 94% [95% confidence interval (CI) 87–100], 13% (95% CI 10–16), 17% (95% CI 14–21) and 78% (95% CI 75–80), and specificities of 88% (95% CI 84–93), 100% (95% CI 99–100), 97% (95% CI 96–99) and 77% (95% CI 75–79), respectively. Pneumococcal attributable proportion was 34% (95% CI 32–37), increasing with age and in men. We estimated that Spn caused one third of CAP. Whole blood *lytA* rt-PCR was more sensitive than SABC; both had low sensitivity and high specificity. Conversely CXR was highly sensitive and reasonably specific; it could be a useful tool for epidemiological studies aiming to define Spn pneumonia incidence across all ages.

## INTRODUCTION

Community-acquired pneumonia (CAP) causes significant morbidity and mortality worldwide, particularly in children aged <5 years [1, 2] and the elderly [3, 4]. *Streptococcus pneumoniae* (Spn) is the leading aetiological agent identified in CAP, causing ~25% of CAP in adults and 8% in young children [5–7]. However, there is currently no adequate gold standard for aetiological diagnosis of CAP: even with the best clinical and laboratory tools, 30–60% of cases have no confirmed aetiology [8, 9].

Numerous tests exist to identify the bacterial aetiology of CAP, but they all suffer limitations. Lung aspirates and pleural fluid cultures are sensitive and specific for bacterial identification, but their collection is invasive and rarely indicated. Sputum and nasopharyngeal swab testing cannot differentiate between pathogens colonizing the upper respiratory tract and those causing lower respiratory tract infections [10]. Blood cultures have a sensitivity of 0–14% [11–13] because of infrequent bacteraemia [5], prior antibiotic use, and inadequate sample volumes, especially in children [13]. For pneumococcal detection, urine antigen testing is more sensitive than blood culture, but false-positive results arise in populations with high nasopharyngeal carriage such as children [14, 15] or patients with recent pneumococcal infection [11]. Polymerase chain reaction (PCR) on blood specimens has been suggested as a potential diagnostic tool with superior sensitivity over blood culture combined with high specificity [16–20] but its validity remains uncertain. The World Health Organization (WHO) developed radiological criteria to standardize the interpretation of paediatric chest radiography (CXR) in clinical trials of *Haemophilus influenzae* type b (Hib) vaccine and pneumococcal conjugate vaccine (PCV). For these trials, primary endpoint pneumonia (PEP) was defined as alveolar consolidation or pleural effusion on CXR, as agreed upon by two of three independent readers [21]. Compared to all severe or hospitalized pneumonia, this WHO-defined outcome is more likely associated with pneumococcus or Hib. For example, a trial in The Gambia found that 9-valent PCV prevented 12% of all severe pneumonia and 37% of PEP [22]. This approach also has limitations: it has been evaluated primarily in children aged <2 years [23] and its sensitivity and specificity are poorly defined. In sum, the lack of a gold standard to diagnose pneumococcal pneumonia makes the evaluation of new laboratory tools for aetiological confirmation challenging.

The PneumoTone study aimed to gather baseline data on pneumococcal meningitis and pneumonia epidemiology in northern Togo, located in the African meningitis belt, to assess the impact of PCV introduction on Spn disease burden across all age groups. In this sub-study, we used latent class analysis (LCA) to assess the diagnostic value of routine tests such as semi-automated blood culture (SABC), CXR, and serum C-reactive protein (CRP), as well as newer tests such as the *lytA* real-time PCR (rt-PCR) on whole blood in diagnosing Spn in CAP patients in northern Togo. We then applied these findings to estimate the proportion of CAP attributable to Spn in our study population.

## METHODS

### Study population

We included all patients residing in Tône or Cinkassé districts, northern Togo, who presented with clinical signs or symptoms of pneumonia, were hospitalized for at least one night at one of the five study sites during 1 May 2010 to 31 October 2013 and provided informed consent to participate.

Togo introduced Hib conjugate vaccine into its routine immunization programme during 2008. Vaccine coverage with three doses of pentavalent vaccine was 81·6% in children aged <24 months at the national level during 2013 [24].

### Data collection

We defined clinical pneumonia, severe pneumonia, PEP, suspected pneumococcal pneumonia, and confirmed pneumococcal pneumonia using PneumoADIP criteria [25]. Following medical examination, data were collected on medical history, including HIV status (self-reported), rhinopharyngitis or pneumonia in the last 2 weeks, and symptoms at admission. We defined sepsis based on clinical signs as in Goldstein *et al*. for children [26] and Bone *et al*. for adults [27]. The following events were considered acute complications: appearance or worsening of cardio-respiratory distress, appearance or worsening of severity criteria, secondary infection, sepsis, organ failure, and death.

### Radiological and laboratory tests

All patients had an antero-posterior CXR obtained on admission, which was digitalized and read off-site by a US paediatrician experienced in reading adult and paediatric CXRs as well as a Togolese general radiologist; a second US paediatrician who participated in the original development of the WHO radiological criteria [21]arbitrated cases where the primary readers had discordant findings. All readers were trained on a standard set of films and used WHO criteria for identifying PEP including presence or absence of alveolar consolidation or pleural effusion and the size of any consolidation. Laboratory tests included two sets of two blood cultures collected 30–90 min apart and inoculated into aerobic and anaerobic BacT/ALERT^®^ bottles (bioMérieux, France) for adults and two blood cultures collected 30–90 min apart cultured in paediatric bottles for children aged <15 years, serum CRP dosage (SECOMAM^®^ spectrophotometer; Secomam, France), and rt-PCR on whole blood to identify Spn (*lytA* gene), Hib (*bexA* gene) and *Staphylococcus aureus* (*etvick* gene) with a Ct cut-off of 35 for positivity [28]. A subset of patients had a nasopharyngeal aspirate collected and tested using the Fast Track Diagnostics Respiratory 21+ multiplex PCR platform (Fast Track Diagnostics, Luxembourg) for viral and bacterial detection.

### Statistical analysis

We used LCA [29] to construct eight models enabling us to categorize patients into two classes interpreted as likely pneumococcal CAP or non-pneumococcal CAP. The models all included laboratory tests (SABC, serum CRP levels, *lytA* rt-PCR) and the presence or absence of PEP on CXR; various combinations of clinical criteria were also included in some models. Local dependence between blood culture and *lytA* rt-PCR was evaluated using a likelihood ratio test comparing models with independent tests to models with tests combined into a single variable. Goodness-of-fit was assessed by parametric bootstrap (2000 simulations). A total of four models with adequate fit were found, and all of them incorporated local dependence between blood culture and *lytA* rt-PCR. The final models included PEP on CXR, blood culture, *lytA* rt-PCR and CRP level, the latter categorized in quartiles or dichotomized with a cut-off of 40 mg/l [10]. Two models also included the occurrence of acute complications. The proportion of CAP attributable to Spn, the sensitivity and specificity of each test, and the receiver operating characteristic (ROC) curve for serum CRP dosage were estimated based on the models’ categorization of likely pneumococcal CAP or non-pneumococcal CAP. The corresponding 95% confidence intervals were assessed by non-parametric bootstrap (2000 simulations). We performed a descriptive analysis of both identified latent classes for the model with good fit that used the smallest number of variables. We also conducted a sensitivity analysis to confirm our *a priori* hypothesis of two latent classes and not more, and to check the respective weight of each of the laboratory and radiological tests in the categorization of cases by the model. Finally, we performed a subgroup analysis using a similar approach on patients with nasopharyngeal aspirate testing done, including an indicator variable for Spn colonization in the LCA models.

Statistical analyses were performed using Stata v. 12 (StataCorp LP, USA), and R v. 3.0.2 (R Foundation for Statistical Computing, Austria) with poLCA and OptimalCutpoints packages.

### Ethics statement

The study followed the ethical principles of the Declaration of Helsinki, recommendations of the French Speaking Epidemiologists Association (ADELF), the International Conference on Harmonization, and the Council for International Organizations of Medical Sciences. The surveillance protocol was approved by National Ethical Committee of Togo and a French ethical review committee. All patients enrolled in surveillance (or their guardians) provided written informed consent.

## RESULTS

Of the 1684 patients with CAP enrolled at one of the study sites from May 2010 to October 2013, 1501 (89·1%) had all tests done and were included in the analysis (Table 1). Of these, 596 (39·7%) had a CXR with PEP, 66 (4·4%) had a blood culture positive for Spn, 113 (7·5%) were positive by *lytA* rt-PCR, and 886 (59·0%) had CRP ⩾40 mg/l. Thirty-two Spn were identified by both blood culture and *lytA* rt-PCR, 81 by PCR only, and 34 by blood culture only. Nasopharyngeal aspirate testing was performed on 838 patients.

**Table 1.** Characteristics of patients hospitalized for community-acquired pneumonia included in the analysis in Tône and Cinkassé districts, northern Togo, 2010–2013

			Age					Severity				
	Total (*n* = 1501)		<5 years (*n* = 216)		⩾5 years (*n* = 1285)			Not severe (*n* = 1139)		Severe (*n* = 362)		
	No.	%	No.	%	No.	%	*P* value	No.	%	No.	%	*P* value
Gender												
Female	651	43·4	88	40·7	563	43·8	0·399	496	43·6	155	42·8	0·807
Male	850	56·6	128	59·3	722	40·7		643	56·5	207	57·2	
Age group, years	64	4·3	64	29·6	0	0·0		36	3·2	28	7·7	**<0·001**
<1	152	10·1	152	70·4	0	0·0		111	9·8	41	11·3	
1–4	233	15·5	0	0·0	233	18·1		189	16·6	44	12·2	
5–14	387	25·8	0	0·0	387	30·1		303	26·6	84	23·2	
15–29	584	38·9	0	0·0	584	45·5		447	39·2	137	37·9	
30–64	81	5·4	0	0·0	81	6·3		53	4·7	28	7·7	
⩾65												
HIV status												
Negative	1131	95·8	122	100·0	1009	95·3	**0·014**	871	95·8	260	95·7	0·868
Positive	50	4·2	0	0·0	50	4·7		38	4·2	12	4·4	
Antecedent of tuberculosis												
No	1313	99·3	146	100·0	1167	99·2	0·609	998	99·5	315	98·8	0·232
Yes	9	0·7	0	0·0	9	0·8		5	0·5	4	1·3	
Antecedent of asthma												
No	1333	97·4	153	98·1	1180	97·3	0·79	1014	97·5	319	95·2	0·592
Yes	36	2·6	3	1·9	33	2·7		26	2·5	10	4·8	
Recent rhinopharyngitis*												
No	940	65·4	126	64·6	814	65·5	0·812	697	64·0	243	69·6	0·055
Yes	498	34·6	69	35·4	429	34·5		392	36·0	106	30·4	
Recent influenza†												
No	1243	87·2	179	95·2	1064	86·0	**<0·001**	949	87·5	294	86·2	0·548
Yes	183	12·8	9	4·8	174	14·1		136	12·5	47	13·8	
Use of antipyretics before consulting												
No	247	17·0	41	19·6	206	16·6	0·278	179	16·2	68	19·7	0·134
Yes	1205	83·0	168	80·4	1037	83·4		927	83·8	278	80·4	
Use of antibiotics before consulting												
No	1087	76·0	137	65·6	950	77·7	**<0·001**	889	80·7	198	60·2	**<0·001**
Yes	344	24·0	72	34·5	272	22·3		213	19·3	131	39·8	
Severe pneumonia												
No	1139	75·9	147	68·1	992	77·2	**0·004**	1139	100·0	0	0·0	
Yes	362	24·1	69	31·9	293	22·8		0	0·0	362	100·0	
Dyspnoea												
No	40	2·7	1	0·5	39	3·0	**0·022**	29	2·6	11	3·0	0·612
Yes	1461	97·3	215	99·5	1246	97·0		1110	97·5	351	97·0	
Tachypnoea												
No	110	7·3	21	9·7	89	6·9	0·145	94	8·3	16	4·4	**0·015**
Yes	1391	92·7	195	90·3	1196	93·1		1045	91·8	346	95·6	
Cough												
No	20	1·3	2	0·9	18	1·4	0·756	13	1·1	7	1·9	0·291
Yes	1481	98·7	214	99·1	1267	98·6		1126	98·9	355	98·1	
Type of cough												
Dry	443	29·9	98	45·8	345	27·2	**<0·001**	348	30·9	95	26·8	0·137
Wet	1038	70·1	116	54·2	922	72·8		778	69·1	260	73·2	
Clinical sepsis												
No	701	46·7	98	45·8	345	27·2	**<0·001**	612	53·7	89	24·6	**<0·001**
Yes	800	53·3	116	54·2	922	72·8		527	46·3	273	75·4	
Hypoxia												
No	1364	91·0	189	87·5	1175	91·6	0·053	1139	100·0	225	62·5	**<0·001**
Yes	135	9·0	27	12·5	108	8·4		0	0·0	135	37·5	
Cyanosis												
No	1497	99·7	215	99·5	1282	99·8	0·463	1139	100·0	358	98·9	**0·003**
Yes	4	0·3	1	0·5	3	0·2		0	0·0	4	1·1	
Lethargy												
No	1243	82·8	183	84·7	1060	82·5	0·421	1139	100·0	104	28·7	**<0·001**
Yes	258	17·2	33	15·3	225	17·5		0	0·0	258	71·3	
Convulsions												
No	1492	99·4	210	97·2	1282	99·8	**<0·001**	1139	100·0	353	97·5	**<0·001**
Yes	9	0·6	6	2·8	3	0·2		0	0·0	9	2·5	
Acute complications												
No	1420	94·6	207	95·8	1213	94·4	0·387	1098	96·4	322	89·0	**<0·001**
Yes	81	5·4	9	4·2	72	5·6		41	3·6	40	11·1	
Death												
No	1471	98·0	209	96·8	1262	98·2	0·183	1129	99·1	342	94·5	**<0·001**
Yes	30	2·0	7	3·2	23	1·8		10	0·9	20	5·5	
PEP on CXR												
No	905	60·3	155	71·8	750	58·4	**<0·001**	728	63·9	177	48·9	**<0·001**
Yes	596	39·7	61	28·2	535	41·6		411	36·1	185	51·1	
Spn identified with blood culture												
No	1435	95·6	214	99·1	1221	95·0	**0·007**	1097	96·3	338	93·4	**0·017**
Yes	66	4·4	2	0·9	64	5·0		42	3·7	24	6·6	
*LytA* rt-PCR positive												
No	1388	92·5	202	93·5	1186	92·3	0·529	1073	94·2	315	87·0	**<0·001**
Yes	113	7·5	14	6·5	99	7·7		66	5·8	47	13·0	
CRP >40 mg/l												
No	615	41·0	104	48·2	511	39·8	**0·02**	503	44·2	112	30·9	**<0·001**
Yes	886	59·0	112	51·9	774	60·2		636	55·8	250	69·1	

HIV, Human immunodefficieny virus; PEP, primary endpoint pneumonia; CXR, chest radiography; Spn, *Streptococcus pneumoniae*; PCR, polymerase chain reaction; CRP, C-reactive protein.

* Recent rhinopharyngitis is defined by a rhinopharyngitis episode in the last 2 weeks.

† Recent influenza is defined by an influenza episode in the last 2 weeks.

*P* values <0·05 are shown in bold.

**Fig. 1.**
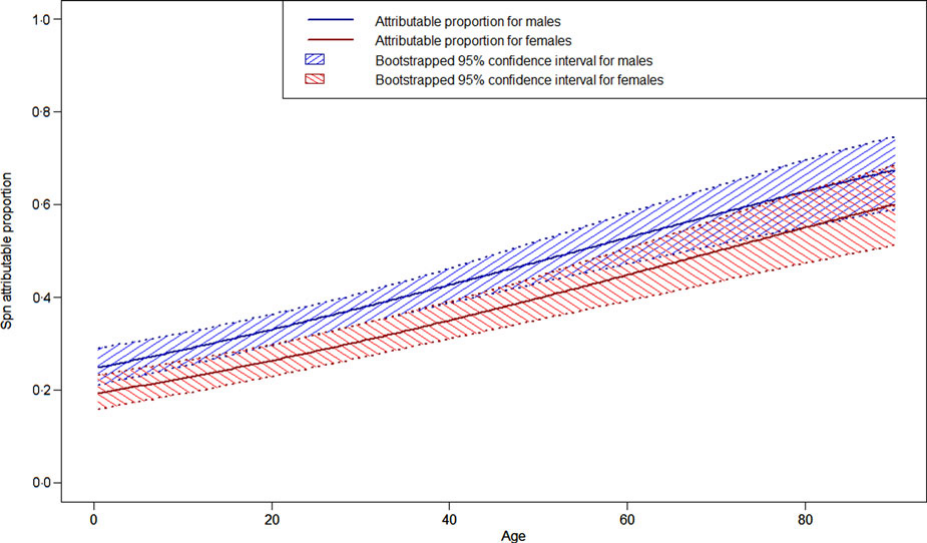
Estimated proportion of community-acquired pneumonia attributable to *Streptococcus pneumoniae* (Spn) by age and by sex in Tône and Cinkassé districts, northern Togo, 2010–2013.

The four final LCA models produced highly consistent results, with kappas for categorizing cases as likely Spn or likely non-Spn CAP from 0·928 to 0·999, so only the model including CXR and the three laboratory tests (with dichotomized CRP) is presented below. The characteristics of the two categories defined by the LCA model pointed to one of them as likely pneumococcal CAP (Table 2). In this category 99·2% of the patients had PEP on CXR, 12·8% had a positive blood culture, 16·1% had a positive *lytA* rt-PCR and 99·6% had CRP>40 mg/l compared to 8·6%, 0%, 3·0% and 37·8%, respectively in the other category (*P* < 0·001 for each comparison). In the 515 likely pneumococcal CAP, four patients had no PEP on CXR but had a positive blood culture or *lytA* rt-PCR and CRP >40 mg/l. Of the 986 likely non-pneumococcal CAP, 85 had PEP on CXR, but 81 of these had negative blood culture and *lytA* rt-PCR and CRP <40 mg/l. Based on the model, Spn was a common aetiology of CAP, causing an estimated 515 cases (34·3%, 95% CI 32·1–37·0), and was more frequent with increasing age (*P* < 0·001) and in male patients (*P* = 0·004), particularly in the 20–40 years age group (Fig. 1). Cases identified as likely pneumococcal CAP were more often severe than likely non-pneumococcal cases with a higher frequency of lethargy, hypoxia, clinical signs of sepsis, and acute complications, and a higher case-fatality ratio (4·5% *vs*. 0·7%, *P* < 0·001). Most of these differences were also observed in children aged <5 years.

**Table 2.** Characteristics of patients with community-acquired pneumonia attributable to *Streptococcus pneumoniae* and attributable to another cause based on the latent classes in Tône and Cinkassé districts, northern Togo, 2010–2013

	Likely non-pneumococcal CAP (*n* = 986)		Likely pneumococcal CAP (*n* = 515)		*P* value	<5 years (*n* = 216)					⩾5 years (*n* = 1285)				
						Likely non- pneumococcal CAP (*n* = 173)		Likely pneumococcal CAP (*n* = 43)		*P* value	Likely non- pneumococcal CAP (*n* = 813)		Likely pneumococcal CAP (*n* = 472)		*P* value
	No.	%	No.	%		No.	%	No.	%		No.	%	No.	%	
Gender															
Female	453	45·9	198	38·5	**0·005**	70	40·46	18	41·9	0·867	383	47·1	180	38·1	**0·002**
Male	533	54·1	317	61·6		103	59·54	25	58·1		430	52·9	292	61·9	
Age group, years															
<1	51	5·2	13	2·5	**<0·001**	51	29·48	13	30·2	0·923	0	0	0	0·0	**<0·001**
1–4	122	12·4	30	5·8		122	70·52	30	69·8		0	0	0	0·0	
5–14	175	17·8	58	11·3		0	0	0	0·0		175	21·5	58	12·3	
15–29	260	26·4	127	24·7		0	0	0	0·0		260	32	127	26·9	
30–64	337	34·2	247	48·0		0	0	0	0·0		337	41·5	247	52·3	
⩾65	41	4·2	40	7·8		0	0	0	0·0		41	5·04	40	8·5	
HIV status															
Negative	768	96·2	363	94·8	0·243	100	100	22	100·0		668	95·7	341	94·5	0·366
Positive	30	3·8	20	5·2		0	0	0	0·0		30	4·3	20	5·5	
Antecedent of tuberculosis															
No	836	99·7	477	98·6	0·068	117	100	29	100·0		761	99·6	406	98·5	0·073
Yes	3	0·3	6	0·7		0	0	0	0·0		3	0·39	6	1·5	
Antecedent of asthma															
No	884	96·7	449	98·7	**0·032**	127	97·69	26	100·0	1	757	96·6	423	98·6	**0·042**
Yes	30	3·3	6	1·3		3	2·31	0	0·0		27	3·44	6	1·4	
Recent rhinopharyngitis*															
No	570	60·1	370	75·7	**<0·001**	99	62·26	27	75·0	0·149	471	59·6	343	75·7	**<0·001**
Yes	379	39·9	119	24·3		60	37·74	9	25·0		319	40·4	110	24·3	
Recent influenza†															
No	819	86·9	424	87·6	0·724	144	94·12	35	100·0	0·213	675	85·6	389	86·6	0·597
Yes	123	13·1	60	12·4		9	5·88	0	0·0		114	14·5	60	13·4	
Use of antipyretics before consulting															
No	146	15·1	101	20·9	**0·006**	35	20·71	6	15·0	0·413	111	13·9	95	21·4	**0·001**
Yes	822	84·9	383	79·1		134	79·29	34	85·0		688	86·1	349	78·6	
Use of antibiotics before consulting															
No	759	79·3	328	69·2	**<0·001**	115	68·45	22	53·7	0·074	644	81·6	306	70·7	**<0·001**
Yes	198	20·7	146	30·8		53	31·55	19	46·3		145	18·4	127	29·3	
Severe pneumonia															
No	786	79·7	353	68·5	**<0·001**	116	67·05	31	72·1	0·526	670	82·4	322	68·2	**<0·001**
Yes	200	20·3	162	31·5		57	32·95	12	27·9		143	17·6	150	31·8	
Dyspnoea															
No	22	2·2	18	3·5	0·149	0	0	1	2·3	0·199	22	2·71	17	3·6	0·367
Yes	964	97·8	497	96·5		173	100	42	97·7		791	97·3	455	96·4	
Tachypnoea															
No	69	7·0	41	93·0	0·497	15	8·67	6	14·0	0·295	54	6·64	35	7·4	0·599
Yes	917	8·0	474	92·0		158	91·33	37	86·1		759	93·3	437	92·6	
Cough															
No	13	1·3	7	1·4	0·558	1	0·58	1	2·3	0·359	12	1·48	6	1·3	1
Yes	973	98·7	508	98·6		172	99·42	42	97·7		801	98·5	466	98·7	
Type of cough															
Dry	348	35·8	95	18·7	**<0·001**	85	49·42	13	31·0	**0·031**	263	32·8	82	17·6	**<0·001**
Wet	625	64·2	413	81·3		87	50·58	29	69·1		538	67·2	384	82·4	
Clinical sepsis															
No	559	56·7	142	27·6	**<0·001**	75	43·35	8	18·6	**0·003**	484	59·5	134	28·4	**<0·001**
Yes	427	43·3	373	72·4		98	56·65	35	81·4		329	40·5	338	71·6	
Hypoxia															
No	909	92·2	455	88·7	**0·025**	150	86·71	39	90·7	0·611	759	93·4	416	88·5	**0·003**
Yes	77	7·8	58	11·3		23	13·29	4	9·3		54	6·64	54	11·5	
Cyanosis															
No	984	99·8	513	99·6	0·611	172	99·42	43	100·0	1	812	99·9	470	99·6	0·558
Yes	2	0·2	2	0·4		1	0·58	0	0·0		1	0·12	2	0·4	
Lethargy															
No	861	87·3	382	74·2	**<0·001**	148	85·55	35	81·4	0·498	713	87·7	347	73·5	**<0·001**
Yes	125	12·7	133	25·8		25	14·45	8	18·6		100	12·3	125	26·5	
Convulsions															
No	979	99·3	513	99·6	0·727	168	97·11	42	97·7	1	811	99·8	471	99·8	1
Yes	7	0·7	2	0·4		5	2·89	1	2·3		2	0·25	1	0·2	
Acute complications															
No	958	97·2	462	89·7	**<0·001**	166	95·95	41	95·4	1	792	97·4	421	89·2	**<0·001**
Yes	28	2·8	53	10·3		7	4·05	2	4·7		21	2·58	51	10·8	
Death															
No	979	99·3	492	95·5	**<0·001**	169	97·69	40	93·0	0·143	810	99·6	452	95·8	**<0·001**
Yes	7	0·7	23	4·5		4	2·31	3	7·0		3	0·37	20	4·2	
PEP on CXR															
No	901	91·4	4	0·8	**<0·001**	155	89·6	0	0·0	**<0·001**	746	91·8	4	0·9	**<0·001**
Yes	85	8·6	511	99·2		18	10·4	43	100·0		67	8·24	468	99·2	
Spn identified with blood culture															
No	986	100·0	449	87·2	**<0·001**	173	100	41	95·4	**0·004**	813	100	408	86·4	**<0·001**
Yes	0	0·0	66	12·8		0	0	2	4·7		0	0	64	13·6	
*LytA* rt-PCR positive															
No	956	97·0	432	83·9	**<0·001**	166	95·95	36	83·7	**0·004**	790	97·2	396	83·9	**<0·001**
Yes	30	3·0	83	16·1		7	4·05	7	16·3		23	2·83	76	16·1	
CRP > 40 mg/l															
No	613	62·2	2	0·4	**<0·001**	104	60·12	0	0·0	**<0·001**	509	62·6	2	0·4	**<0·001**
Yes	373	37·8	513	99·6		69	39·88	43	100·0		304	37·4	470	99·6	

HIV, Human immunodefficieny virus; PEP, primary endpoint pneumonia; CXR, chest radiography; Spn, *Streptococcus pneumoniae*; PCR, polymerase chain reaction; CRP, C-reactive protein.

* Recent rhinopharyngitis is defined by a rhinopharyngitis episode in the last 2 weeks.

† Recent influenza is defined by an influenza episode in the last 2 weeks.

*P* values <0·05 are shown in bold.

Table 3 presents the sensitivity and specificity of PEP, blood culture, and *lytA* rt-PCR estimated by the LCA model. Based on these data, PEP had the highest sensitivity yet remained reasonably specific for detecting likely Spn aetiology. *lytA* rt-PCR had higher sensitivity than SABC (*P* = 0·006) and the combination of *lytA* rt-PCR plus SABC had higher sensitivity than *lytA* rt-PCR alone (*P* = 0·027). The serum CRP cut-off optimizing both sensitivity and specificity based on the analysis of the area under the ROC curve was 71·2 mg/l (95% CI 67·6–75·1). Comparison of the diagnostic values of each test after estimates done separately in children aged <5 years *vs*. older patients found that the sensitivity and specificity of PEP and *lytA* rt-PCR did not differ significantly across the two age groups, whereas blood culture was less sensitive in young children (4·4% *vs*. 13·6%, *P* = 0·031) but similarly specific (100% for both groups).

**Table 3.** Diagnostic values of blood culture, lytA rt-PCR, CRP level, and chest radiography to identify likely pneumococcal pneumonia as categorized by the latent class analysis

	Sensitivity (%)	95% CI	Specificity (%)	95% CI
Blood culture	12·9	9·9–16·4	100·0	99·9–100
*lytA* rt-PCR	17·1	13·6–20·8	97·4	96·2–98·5
Blood culture or *lytA* rt-PCR	23·8	19·6–28·0	97·4	96·2–98·5
CRP > optimal cut-off	77·7	75·1–80·0	77·2	74·9–79·3
PEP on CXR	93·7	87·3–100	88·1	84·3–92·5

CI, Confidence interval; PCR, polymerase chain reaction; CRP, C-reactive protein; PEP, primary endpoint pneumonia; CXR, chest radiography.

Of the 838 patients with a nasopharyngeal aspirate done, 230 (27·5%) had Spn carriage identified. Pneumococcal nasopharyngeal carriage was more frequent in children age <5 years compared to older persons (50·5% *vs*. 24·3%, *P* < 0·001), in patients with PEP compared to no PEP (38·9% *vs*. 20·6%, *P* < 0·001), with a positive compared to a negative blood culture for Spn (71·0% *vs*. 25·8%, *P* < 0·001), with a positive compared to a negative *lytA* rt-PCR (47·6% *vs*. 26·4%, *P* = 0·004), and with CRP ⩾40 mg/l compared to lower CRP levels (35·6% *vs*. 19·2%, *P* < 0·001). None of the LCA models including nasopharyngeal aspirate results had good statistical fit. Using the LCA-defined categories, Spn nasopharyngeal carriage was more frequent in likely pneumococcal CAP (255 patients) than non-pneumococcal CAP (583 patients) (42·4% *vs*. 20·9%, *P* < 0·001) and had a positive predictive value (PPV) of 47% (108/230) and a negative predictive value (NPV) of 76% (461/608).

## DISCUSSION

In this study, we used an innovative mathematical approach to investigate the diagnostic value of radiological and laboratory findings to identify Spn as the causal agent for CAP, and then estimate its contribution to CAP burden in the population of northern Togo. We calculated that in all age groups and in the context of Hib conjugate and whole-cell pertussis vaccine use in national infant immunization schedules, 34·3% of hospitalized CAP cases were likely attributable to pneumococcus. This proportion was higher in men than women and increased with age. Cases categorized as likely pneumococcal CAP were more severe, were associated with more complications, and had a higher case-fatality ratio than cases categorized as non-pneumococcal CAP, consistent with what has been described elsewhere [30, 31]. PEP on CXR was the most sensitive test for identifying likely Spn cases. Specificity of PEP was lower than that of microbiological tools, but was still very high at nearly 90%. *LytA* rt-PCR on whole blood was superior to blood culture in terms of sensitivity and both were highly specific.

Our estimate of the proportion of CAP likely attributable to Spn was consistent with the literature. Spn was estimated to cause 25% of CAP in a meta-analysis of studies of European adults [5]. In Kenyan adults, 46% of CAP was attributable to Spn in a prospective study that also used LCA [32], compared to 38·0% in patients aged ⩾18 years in our study. The increasing prevalence of likely Spn with age is also compatible with the fractions attributable to Spn observed in the literature including 8% in children [7], 25% in European adults [5], and close to 50% in the elderly [33].

Our estimate of the diagnostic value of PEP did not differ significantly between children aged <5 years and patients aged ⩾5 years, despite WHO CXR interpretation guidelines having been developed and validated mainly for children aged <2 years [25]. For comparative purposes, we calculated the proportion of Spn in CXR-confirmed CAP cases (equivalent to the positive PPV of PEP on CXR) in young children in a trial of 9-valent PCV from The Gambia [22]. This was done by dividing the vaccine efficacy (VE) of PCV against PEP by the VE against all invasive pneumococcal disease, yielding a PPV estimate of 74%. The NPV of PEP was 90% based on a similar calculation. These estimates are consistent with our findings of a PPV and NPV of 85·7% and 99·6%, respectively, and support the importance of PEP on CXR for identifying likely Spn pneumonia cases in all age groups, although further data are needed to strengthen the methodology for older children and adults. It is important to note that these measures of diagnostic value refer to the WHO standard methodology of CXR interpretation, and are therefore unlikely to be applicable to routine clinical care in a resource-poor setting.

*lytA* rt-PCR on whole blood was superior to blood culture in terms of sensitivity. Previous studies in mice [17, 18] and humans [34, 35] have shown PCR on whole blood to have a higher sensitivity, although comparing earlier results to our findings is difficult because of different study designs, analytical approaches, blood fraction used for rt-PCR, and small sample sizes. The sensitivity we found for blood culture was low and consistent with other studies [13]. A significantly lower sensitivity was found in children aged <5 years, whereas *lytA* rt-PCR sensitivity did not vary significantly between age groups. Although *lytA* rt-PCR was more sensitive than blood culture, using a combination of both tests significantly improved the overall sensitivity, illustrating their complementary value for aetiological confirmation of cases. *lytA* rt-PCR specificity was high but not 100%, which could be explained by cross-reactivity with other bacteria such as *Streptococcus mitis* [36].

The lower frequency of recent rhinopharyngitis in likely pneumococcal CAP and the similar frequency of recent influenza in both groups is counter-intuitive, as viral infections (particularly influenza) are known to be associated with pneumococcal pneumonia [37]. However, recent rhinopharyngitis and influenza were both self-reported and may therefore be subject to misclassification. In addition, the role of influenza as a precursor of pneumococcal pneumonia has been shown in the case of severe pandemic influenza [38] but is less well established in inter-pandemic periods and for mild disease. Finally, our analysis of nasopharyngeal aspirate data found no association between viral infection in general (or influenza infection in particular) and pneumococcal colonization and a negative association between viral infection and pneumococcal bacteraemia, suggesting that these interactions are not straightforward [39].

Pneumococcal nasopharyngeal carriage could not be included in the LCA models. The subgroup analysis revealed that colonization has poor predictive value for likely Spn CAP. This is consistent with the observation that Spn carriage does not necessarily predict Spn CAP [40].

Our study had several limitations. The final LCA models included only four tests and two of them were not independent. Sensitivity analysis revealed that the LCA models relied heavily on PEP for categorization of patients; the other tests identified only a small number of additional likely Spn cases in patients who were PEP-negative. Pneumococcal nasopharyngeal colonization findings were not included in the LCA models because they led to poor fit. However, the addition of other diagnostic tools such as urine antigen detection tests for pneumococcal capsular polysaccharide, which were not done in our study, might have further strengthened our analysis.

The LCA approach offers the opportunity to limit the bias inherent to the use of imperfect tests in diagnostic evaluations. Together, the large and representative sample of cases, the similarity of findings in the four final LCA models, and the consistency with the literature all support the validity of our results. We estimated that Spn may cause about one third of CAP cases in our population and plays an important role in both childhood and adult disease. We provide data that PEP on CXR may have a role in monitoring PCV impact in older populations. We have continued to monitor hospitalized pneumonia trends in this meningitis belt population after the 2014 introduction of PCV13 into the routine immunization programme and plan to use LCA to measure the proportion of CAP likely due to Spn in the post-vaccine introduction period and improve estimations of vaccine impact.
